# Neighborhood-level disadvantage and lifestyle-based supportive care in head and neck cancer: insights from the Midwestern United States

**DOI:** 10.20935/acadonco8104

**Published:** 2026-01-20

**Authors:** Monica A. Wagner, Brandon Vu, Charles Djordjevic, Naji Ayyash, Ravi K. Kyasaram

**Affiliations:** 1 Frances Payne Bolton School of Nursing, Case Western Reserve University, Cleveland, OH, USA.; 2 Case Comprehensive Cancer Center, Case Western Reserve University School of Medicine, Cleveland, OH, USA.; 3 University Hospitals Seidman Cancer Center, Cleveland OH, USA.

**Keywords:** head and neck neoplasms, neighborhood disadvantage, survivorship, health disparities, lifestyle factors, supportive care

## Abstract

Survivorship care in head and neck cancer (HNC) is complex, influenced by tumor site, treatment modality, and social determinants of health. Neighborhood-level factors may affect cancer outcomes, yet the impact of neighborhood disadvantage on HNC survival remains underexplored. This study examined the association between neighborhood disadvantage and overall survival in HNC survivors. We conducted a retrospective cohort study of patients diagnosed with HNC (*n* = 440) at a single institution (2013–2023). Neighborhood disadvantage was measured using the Area Deprivation Index (ADI) national percentile and stratified into quintiles. Clinical, demographic, and treatment data were extracted from electronic health records. Overall survival was analyzed using Kaplan–Meier curves and the Cox proportional hazards model. Multiple linear regression identified predictors of ADI. Significant differences across ADI quintiles were observed for age at diagnosis (*p* = 0.025), HPVp16 status (*p* < 0.001), and comorbidity count (*p* = 0.028). In multivariable regression, only age (*p* = 0.04) and HPVp16 status (*p* < 0.001) remained significant. Median survival did not differ significantly; however, survival status at study end was notable, with >50% of participants in the most disadvantaged quintile deceased or lost to follow-up. Kaplan–Meier curves demonstrated variation in survival by ADI quintile. Neighborhood disadvantage influences HNC survivorship. Integrating community-level and lifestyle-based interventions (e.g., nutrition education, physical activity promotion, stress reduction, and behavioral health support) into survivorship care may mitigate effects of social inequities. Incorporating neighborhood context into survivorship planning may improve outcomes and promote equity in HNC care.

## 1. Introduction

Head and neck cancer (HNC) accounts for 3–4% of all cancers in the United States, with approximately 70,000 new cases reported annually [1, 2]. Despite advances in multimodal treatment (including surgery, radiation, chemotherapy, and immunotherapy) HNC remains associated with substantial morbidity and mortality [3, 4]. Survivorship is often complicated by functional impairments, psychological distress, and chronic comorbidities that create variability in long-term outcomes [5, 6]. Notably, disparities in HNC outcomes have been increasingly linked to social determinants of health, including socioeconomic status, access to care, and neighborhood-level disadvantage [7–10].

The Area Deprivation Index (ADI) is a validated measure of neighborhood-level disadvantage that incorporates various socioeconomic indicators [11]. While the ADI has been associated with survival disparities in multiple cancer types, its link to HNC survivorship is underexplored [12–17]. Given the complex interplay between biological, clinical, and social factors in HNC, understanding how neighborhood context can influence outcomes is important for developing equitable, patient-centered care strategies.

Emerging evidence suggests that neighborhood disadvantage may impact not only access to care and treatment adherence but also biological and psychosocial factors such as comorbidity burden, stress physiology, and quality of life. For example, higher ADI scores have been linked to increased psychological distress, reduced physical and social-emotional functioning, and delayed initiation of guideline-adherent therapy in HNC populations [18–21]. These findings underscore the importance of considering upstream social and environmental factors when evaluating cancer outcomes.

This study contributes to a growing body of literature examining how social and environmental factors shape cancer trajectories. By exploring the relationship between neighborhood-level disadvantage and survivorship in HNC, we aim to highlight the importance of integrating social context into survivorship care. Understanding these broader influences can inform more equitable approaches to treatment, recovery, and long-term support. The purpose of our study is to examine the association between neighborhood disadvantage and overall survival outcomes in HNC.

This study supports continuing efforts to incorporate social determinants into survivorship care and emphasizes the need for community-informed strategies to promote equitable care for HNC patients.

## 2. Materials and methods

### 2.1. Study population

This retrospective study included individuals newly diagnosed with HNC at University Hospitals Cleveland Medical Center between 2013 and 2023. Eligible patients were identified through the institutional cancer registry. Standardized data on cancer staging were extracted from the registry records. Detailed clinical and treatment information, including patient demographics, medical history, prescribed medications (with specific attention to opioid prescriptions), chemotherapy, radiation therapy, immunotherapy, surgical interventions and comorbidity burden, were obtained through electronic health record review. Comorbidity burden was quantified using the National Cancer Institute Comorbidity Index [22]. Opioid prescription data were collected for a 24-month window spanning from 12 months prior to diagnosis through 12 months post-diagnosis. This study was conducted according to the guidelines of the Declaration of Helsinki and approved by the Institutional Review Board at University Hospitals Cleveland Medical Center, Cleveland, OH (STUDY20250308).

### 2.2. Inclusion and exclusion criteria

Participants were eligible for inclusion if they were aged 18 years or older, had no prior history of cancer, and were newly diagnosed with HNC of the oral cavity or oropharynx. All patients received curative-intent treatment, including surgery, chemotherapy, radiation therapy, immunotherapy, or a multimodal combination. All cancer stages were included and classified according to the American Joint Commission on Cancer TNM staging system [23]. Exclusion criteria included patients with primary tumor sites outside the oral cavity or oropharynx, those who did not receive any form of treatment, and individuals who did not survive at least six months post-diagnosis, to exclude patients receiving palliative care only.

### 2.3. Variables

The primary exposure for this study was neighborhood-level disadvantage, measured using the national percentile of the Area Deprivation Index (ADI). ADI data were obtained via the University of Wisconsin Neighborhood Atlas mapping tool, using the participants’ nine-digit zip codes [11]. The ADI ranks neighborhoods based on census block groupings and incorporates indicators such as income, education, employment, and housing quality. National ADI percentiles range from 1 (least disadvantaged) to 100 (most disadvantaged) and were used in this study to enhance generalizability. Participants were categorized into quintiles based on their ADI percentile: Q1 (1–20), Q2 (21–40), Q3 (41–60), Q4 (61–80), and Q5 (81–100).

The primary outcome was overall survival, defined as the time from date of diagnosis to either the documented date of death or the end of study, whichever occurred first. Patients who were alive at the end of follow-up were censored at their last known date of contact. Survival time was calculated in months. Event status was coded as ‘1’ for death and ‘0’ for censored observations. This definition aligns with standard practice in survival analysis and ensures consistency across similar studies. All survival times and event indicators were derived from electronic health records and verified against institutional cancer registry data to maintain accuracy.

Sociodemographic and clinical characteristics were extracted from the electronic health record. Sociodemographic variables included age (in years), sex (female, male), race (White, Other), opioid prescription before and/or after diagnosis (yes, no), and comorbidity count (0, 1, 2, ≥3). Clinical variables included tumor stage (T1/T2 vs. T3/T4), cancer subtype (oral cavity or oropharyngeal), treatment modality (surgery alone, surgery with adjuvant therapy, nonsurgical), and HPVp16 immunohistochemistry status (positive, negative) for patients with oropharyngeal cancer.

Opioid prescription rates were included as a covariate to explore potential associations between neighborhood-level disadvantage and pain management practices. Opioid use is a relevant supportive care measure in HNC, as disparities in pain control may reflect broader inequities in treatment and quality of life.

### 2.4. Statistical analysis

Descriptive statistics were generated using the ‘CreateTableOne’ function in R (v4.1.2) for the overall cohort stratified by ADI quintile. Continuous variables were assessed for normality using the Shapiro–Wilk test. Based on distribution, either one-way ANOVA (for normally distributed data) or Kruskal–Wallis test (for non-parametric data) was applied. Categorical variables were analyzed using chi-square tests or Fisher’s exact tests where appropriate. A *p*-value threshold of <0.05 was used to determine statistical significance. Variables found to be significantly different between ADI quintiles in the preliminary analyses were included in a multiple linear regression model to assess their relationship with ADI as a continuous outcome (national percentile). Predictor variables included age at diagnosis, HPVp16 status, and comorbidity count.

Kaplan–Meier survival curves, stratified by ADI quintile, were generated to explore overall survival. Differences in survival distribution across quintiles were assessed using the log-rank test. Median and interquartile ranges (IQR) were calculated for each group to summarize central tendency and variability. The number of participants at risk was displayed at regular intervals beneath the survival curves. To estimate the association between neighborhood-level disadvantage and overall survival, a Cox proportional hazards model was fitted using ADI quintile as the primary exposure. The model included survival time (in months) and event status (death = 1, censored = 0) as outcome variables. Hazard ratios and 95% confidence intervals were calculated for each quintile, using Q1 (least disadvantaged) as the reference group. Statistical significance was evaluated using the Wald test, likelihood ratio test, and score (log-rank) test. Because 122 observations (27.7% of the sample) have missing covariate data, we used complete case analysis rather than imputation, as imputation in survival models requires strong assumptions about missingness mechanism and could introduce bias. All survival analyses were conducted using the ‘survival’ and ‘survminer’ packages in R.

## 3. Results

A total of 440 patients met the inclusion criteria to be included in this study. Descriptive statistics found significant differences across ADI quintiles for age at diagnosis (*p* = 0.025), HPVp16 status (*p* < 0.001), and comorbidity count (*p* = 0.028; Table 1). The majority of participants were non-Hispanic, white (94.4%) and male (75.9%). Trends toward significance were noted for treatment modality (*p* = 0.056) and opioid prescription prior to diagnosis (*p* = 0.079). Analysis of survival outcomes demonstrated a decreasing median survival time and end-of-study survival status across increasing ADI, reflecting a gradual worsening of survival with increasing neighborhood disadvantage (Table 2). Although differences in median survival across quintiles did not reach statistical significance, the distribution of survival status at study end was significantly different (*p* = 0.002), with a higher proportion of deceased or lost to follow-up individuals in the most disadvantaged quintile (Q5). These findings support the hypothesis that neighborhood deprivation is associated with poorer survival outcomes, even when differences in median survival are not statistically significant.

To further examine factors associated with higher neighborhood-level deprivation, a multiple linear regression model was fitted with ADI national percentile as the outcome variable. Predictor variables included age at diagnosis, HPVp16 status, and comorbidity count. The model was statistically significant (*p* < 0.001), though it explained only a modest proportion of the variance (adjusted R^2^ = 0.067). Age at diagnosis (*p* = 0.04) and HPVp16 status (*p* < 0.001) were significant predictors. Specifically, older age and HPVp16 positivity were associated with lower ADI percentile, suggesting that patients with these characteristics may reside in less socioeconomically disadvantaged areas.

**Figure 1 •:**
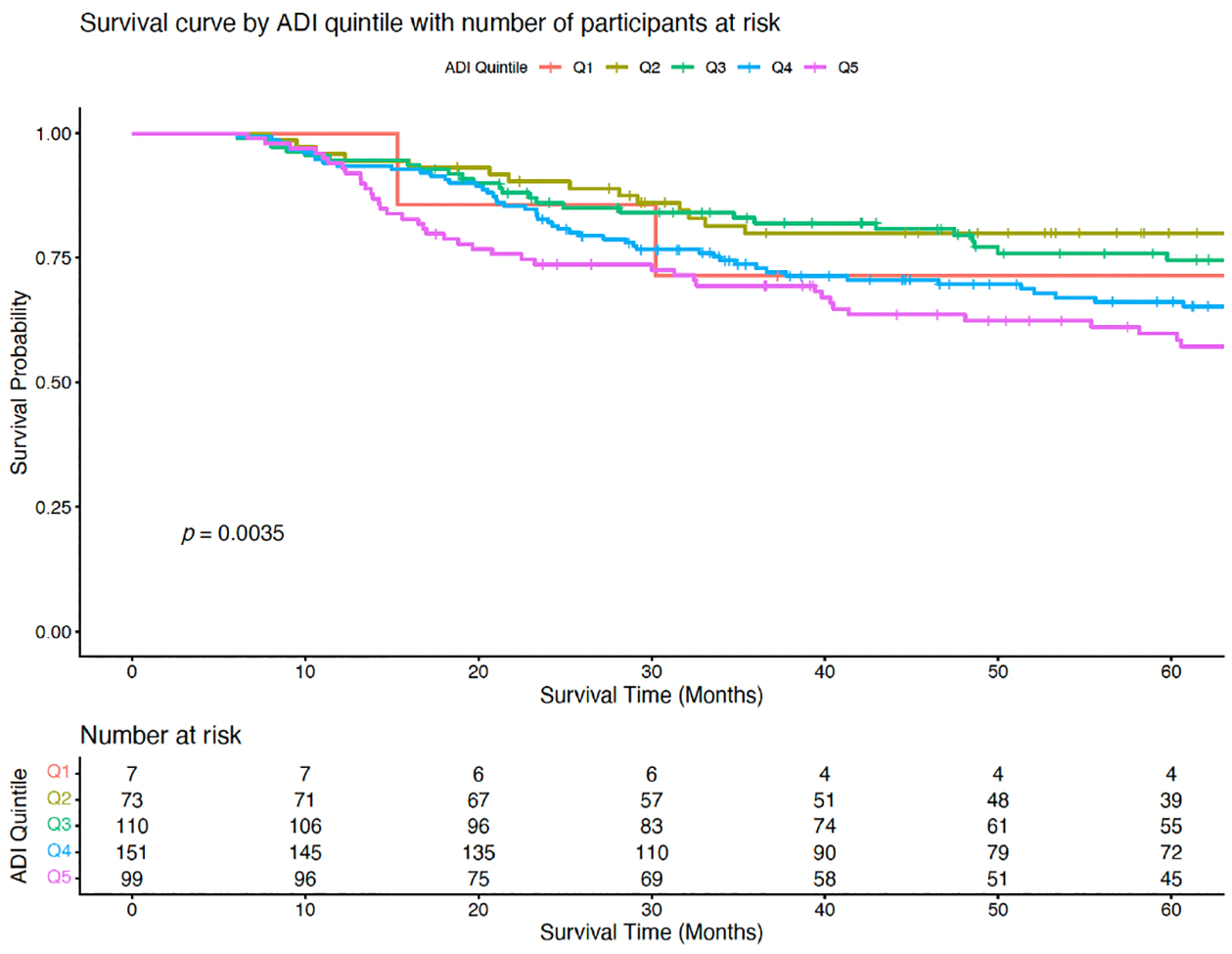
Survival curve showing the survival for each quintile with number at risk. Patients living in most disadvantaged quintile (Q5) experienced the steepest decline in survival while those living in the least disadvantaged areas had a greater survival probability. ADI: Area Deprivation Index.

Kaplan–Meier survival curves revealed significant differences in overall survival across ADI quintiles (Figure 1). Patients in the most socioeconomically deprived quintile (Q5) experienced the steepest decline in survival, with only 45 (45%) remaining at risk by 60 months. In contrast, the least deprived group (Q1) retained 4 participants (57.1%), and the next least deprived group (Q2) retained 39 (53%). A log-rank test comparing survival distributions across quintiles was statistically significant (*p* = 0.0035), indicating that overall survival differed meaningfully by ADI level.

To further evaluate the impact of clinical and demographic covariates on survival, a multivariable Cox proportional hazards model was fitted, adjusting for age at diagnosis, HPVp16 status, and comorbidity burden. The model included 440 patients, with 163 observed events (deaths); however, 122 observations were excluded due to missing data. None of the individual covariates were statistically significant predictors of survival. However, the overall model was statistically significant (Likelihood Ratio test, *p* < 0.001; Wald Test, *p* < 0.001, Score Test, *p* < 0.001). The Concordance Index for the model was 0.687 (SE = 0.021) indicating moderate ability to distinguish between patients with different survival outcomes.

## 4. Discussion

This study examined the relationship between neighborhood-level disadvantage and overall survival outcomes in patients with HNC treated within a large health system in the Midwestern United States. Our findings demonstrate that patients residing in more disadvantaged neighborhoods experienced poorer survival outcomes, consistent with prior evidence linking social determinants of health to cancer prognosis. These results underscore the persistent influence of structural and contextual factors on treatment outcomes, even in settings with standardized clinical care. While the primary focus of this analysis was observational, we also reviewed evidence-based strategies from the literature to identify potential approaches for mitigating these disparities. Lifestyle-based supportive care interventions (e.g., nutrition counseling, physical activity programs, and smoking cessation) have been proposed as promising strategies to improve the quality of life and treatment adherence, particularly in populations facing socioeconomic challenges. These interventions were not implemented or evaluated in the current study; rather, they are highlighted as future directions to complement clinical care and address the multifactorial nature of survival disparities. By integrating our findings with existing evidence, we aim to provide a foundation for subsequent research that combines epidemiologic insights with targeted interventions.

Findings of this study suggest that ADI scores are associated with both survival status and clinical/demographic characteristics, highlighting the importance of social determinants of health in cancer survivorship. Multiple linear regression analysis revealed that younger age and HPVp16 negative status were associated with a higher ADI, suggesting that these patients may be more likely to reside in a socioeconomically disadvantaged neighborhood, reinforcing the need to consider neighborhood context when evaluating patient risk and tailoring survivorship care.

Survival analysis further highlighted the impact of neighborhood-level disadvantage. Kaplan–Meier curves revealed a difference in survival across ADI quintiles, with patients in the most deprived quintile (Q5) exhibiting the steepest decline in survival over time. Although differences in median survival did not reach statistical significance, the distribution of final survival status differed significantly across quintiles, with Q5 showing the highest proportion of deceased or lost to follow-up individuals. These findings are consistent with prior research linking neighborhood disadvantage to poorer cancer outcomes, potentially driven by disparities in access to care, treatment adherence, and comorbidity burden [7, 9, 10, 24–32].

The adjusted Cox proportional hazard model, which included age at diagnosis, HPVp16 status, and comorbidity count, did not identify any individual covariates as statistically significant predictors of survival. However, the overall model was statistically significant, and the concordance index indicated moderate predictive discrimination. These results suggest that traditional clinical variables may not fully account for survival differences. The inclusion of socioeconomic context, such as neighborhood-level disadvantage, may enhance model performance by capturing unmeasured factors that influence outcomes [25].

Although opioid prescription rates were not significantly different across ADI quintiles, there was a trend toward significance in receipt of opioids prior to diagnosis, suggesting potential disparities in pain management or access to supportive care. This finding warrants further investigation, as opioid prescribing practices may reflect broader systemic inequities in symptom control and healthcare access. While this analysis did not evaluate the appropriateness of prescribing or patient adherence, the inclusion of opioid data offers a preliminary view of how treatment-related factors intersect with neighborhood disadvantage. Patients residing in more disadvantaged neighborhoods may face barriers to timely pain assessment, continuity of care, or access to non-opioid alternatives, which could influence both quality of life and treatment adherence [33, 34]. Moreover, the intersection of neighborhood disadvantage and opioid use raises important considerations for survivorship planning, particularly in the context of the opioid epidemic and its disproportionate impact on vulnerable populations [34–36]. Future research should explore whether neighborhood-level disadvantage contributes to differential patterns of opioid use, misuse, or under-treatment of pain in HNC populations and whether targeted interventions could improve equitable access to comprehensive symptom management.

Many of these disparities may be mitigated through modifiable lifestyle factors and targeted interventions. Strategies such as smoking cessation, improved nutrition, physical activity promotion, and management of comorbid conditions (e.g., cardiovascular disease, diabetes) have demonstrated benefits in cancer outcomes and may be especially impactful in socioeconomically disadvantaged populations [37–41]. However, these lifestyle factors are often influenced by environmental and social barriers, including food insecurity, limited access to safe recreational spaces, and reduced availability of preventative healthcare services [38, 42, 43]. Addressing these barriers through community-based health promotion initiatives and policy-level interventions can help reduce the burden of disease in neighborhoods experiencing higher levels of disadvantage.

Stress reduction is another important modifiable lifestyle factor. Chronic stress has been associated with immune dysregulation, increased inflammation, and poorer treatment response, and it may be more prevalent among individuals living in disadvantaged environments [44–47]. Integrating stress-reduction strategies (e.g., mindfulness, yoga, behavioral health support) into survivorship care may enhance quality of life and potentially improve clinical outcomes [32, 48, 49]. These interventions can be delivered through accessible community venues such as libraries, churches, and community health centers [50].

A table of community-level interventions that outlines a range of actionable strategies aligned with issues relevant to HNC survivorship has been provided (Table 3). Examples include community gardens, mobile produce markets, and culturally tailored nutrition education to improve dietary quality; free or low-cost exercise programs and walking groups to promote physical activity and social cohesion; mindfulness classes and behavioral health integration to address insomnia, anxiety, and depression; peer mentorship programs and community-based survivorship clinics to enhance care navigation and psychosocial support; cancer advocacy training and mobile survivorship units to improve patient engagement and follow-up; tobacco cessation and alcohol reduction campaigns tailored to HNC populations; non-emergency medical transportation and telehealth services to overcome geographic and mobility barriers; and enhancements to the built environment and local policy advocacy to support healthy living [40, 51–56].

Integrating social risk screening into survivorship care and tailoring supportive services based on neighborhood-level data may help reduce cancer outcome disparities. For instance, identification of patients from high ADI areas could prompt timely referrals to social work, financial counseling, or survivorship resources. These strategies reflect a growing recognition that addressing social determinants is essential for achieving equity in cancer care [57].

Findings of this study highlight the multifaceted role of neighborhood-level disadvantage in shaping cancer trajectories. The observed associations between ADI and both clinical markers and survival outcomes suggest that neighborhood-level disadvantage may influence cancer progression through interconnected social and biological pathways [58]. Future research should explore mechanisms linking disadvantage to tumor biology, stress physiology, and treatment response. Additionally, interventions should prioritize equity-focused strategies to mitigate disparities in HNC care.

### 4.1. Limitations

This study has several limitations that should be considered when interpreting the findings. First, this study focused exclusively on patients with oral cavity and oropharyngeal cancer (which are most treated at our institution), excluding other HNC subtypes. This may limit the broader application of the results. Additionally, the study population was drawn from the Midwestern United States, which may affect generalizability, as neighborhood characteristics and resource distribution can vary regionally. The dataset also lacked key behavioral risk factors such as smoking and alcohol use, which are known contributors to HNC development. However, the primary aim of this study was not to examine etiologic stressors leading to head and neck cancer but rather to explore community-based interventions that may reduce stress and improve survivorship outcomes. Another limitation is the absence of residential history data; we were unable to determine how long participants had lived in their respective neighborhoods, which may influence the cumulative impact of socioeconomic deprivation. Also, this study did not assess lifestyle-based supportive care interventions because the analysis was retrospective and relied on existing clinical and demographic data rather than intervention-specific measures.

Group sizes across ADI quintiles were uneven, with particularly small sample sizes in the first quintile (Q1), potentially limiting statistical power and precision in comparative analysis. Additionally, HPVp16 status was only available for patients with oropharyngeal cancer, restricting its utility as a clinical marker across the full cohort. In light of these limitations, this study offers valuable insights into the relationship between neighborhood-level disadvantage and survival outcomes, and underscores opportunities for targeted, community-level interventions to address disparities in HNC care.

### 4.2. Strengths

Despite its limitations, this study offers several notable strengths. It examines the relationship between neighborhood-level disadvantage and overall survival outcomes in HNC. By focusing on oral cavity cancer and oropharyngeal cancer, the study targets two clinically and biologically distinct subtypes, enhancing the relevance of findings to precision survivorship strategies. The use of national ADI provides a validated, multidimensional measure of neighborhood disadvantage, allowing for analysis of social determinants of health. Importantly, the study shifts the lens from individual-level risk factors to community-level influences, offering a novel perspective that aligns with emerging public health priorities. As a preliminary investigation, these findings help identify key directions for future research and intervention, particularly those aimed at modifying social and behavioral factors to make survivorship care more equitable and accessible. Ultimately, this work underscores the need for further studies to validate and expand upon these results, ultimately informing targeted, community-based strategies to reduce disparities in HNC outcomes.

## 5. Conclusions

This study demonstrates an association between neighborhood-level disadvantage and overall survival among patients with HNC in the Midwestern United States. While our findings align with prior research showing that social determinants of health influence cancer outcomes, this analysis adds regional evidence from a large health system over a 10-year period. Importantly, this study was observational and did not evaluate lifestyle-based supportive care interventions, which we acknowledge as a critical next step in addressing disparities. Further research should incorporate and compare outcomes from programs that actively implement lifestyle modifications and supportive care strategies to determine their effectiveness in mitigating the impact of neighborhood disadvantage. By highlighting the persistent survival gap associated with socioeconomical context, our findings underscore the need for targeted interventions and policy efforts aimed at reducing structural barriers to equitable cancer care.

## Figures and Tables

**Table 1•:** Characteristics of head and neck cancer patients by quintile.

Variable	Q1 1–20 (*n* = 7)	Q2 21–40 (*n* = 73)	Q3 41–60 (*n* = 110)	Q4 61–80 (*n* = 151)	Q5 81–100 (*n* = 99)	*p*-value
Age, Mean (SD), Years	68.71 (11.28)	61.29 (9.96)	63.84 (9.69)	62.03 (10.29)	60.09 (8.88)	0.025
**Sex**						0.090
Male	5 (71.4)	63 (86.3)	86 (78.2)	105 (69.5)	75 (75.8)	
Female	2 (28.6)	10 (13.7)	24 (21.8)	46 (30.5)	24 (24.2)	
**Race/Ethnicity** ^a^						0.079
Non-Hispanic, White	7 (100)	69 (94.5)	107 (97.3)	142 (94.0)	77 (77.8)	
Non-White	0 (0)	4 (5.5)	3 (2.7)	9 (6.0)	22 (22.2)	
**HNC Subtype**						0.103
Oral Cavity	6 (85.7)	31 (42.5)	55 (50.0)	70 (46.4)	56 (56.6)	
Oropharyngeal	1 (14.3)	42 (57.5)	55 (50.0)	81 (53.6)	43 (43.4)	
Tumor Stage	(*n* = 6)	(*n* = 63)	(*n* = 83)	(*n* = 124)	(*n* = 78)	0.957
T1/T2	1 (16.7)	21 (33.3)	28 (33.7)	39 (31.5)	30 (38.5)	
T3/T4	5 (83.3)	42 (66.7)	55 (66.3)	85 (68.5)	48 (61.5)	
**Treatment Modality**						0.056
Surgery Alone	2 (28.6)	8 (11)	33 (30.0)	50 (33.1)	29 (29.3)	
Surgery with Adjuvant Therapy	3 (42.9)	35 (47.9)	38 (34.5)	53 (35.1)	43 (43.4)	
Nonsurgical	2 (28.6)	30 (41.1)	39 (35.5)	48 (31.8)	27 (27.3)	
**HPVp16 Status**						<0.001
Positive	4 (57.1)	62 (84.9)	80 (72.7)	92 (60.9)	47 (47.5)	
Negative	3 (42.9)	11 (15.1)	30 (27.3)	59 (39.1)	52 (52.5)	
**Receipt of Opioid Prior to Diagnosis**						0.079
Yes	0 (0)	5 (6.8)	4 (3.6)	13 (8.6)	1 (1.0)	
No	7 (100)	68 (93.2)	106 (96.4)	138 (91.4)	98 (99.0)	
**Receipt of Opioid After Diagnosis**						0.129
Yes	6 (85.7)	53 (72.6)	66 (60.0)	97 (64.2)	73 (73.7)	
No	1 (14.3)	20 (27.4)	44 (40.0)	54 (35.8)	26 (26.3)	
**Comorbidity Count**						0.028
0	3 (42.9)	28 (38.4)	43 (39.1)	58 (38.4)	22 (22.2)	
1	0 (0)	20 (27.4)	27 (24.5)	38 (25.2)	29 (29.3)	
2	3 (42.9)	5 (6.8)	24 (21.8)	26 (17.2)	22 (22.2)	
≥3	1 (14.3)	20 (27.4)	16 (14.5)	29 (19.2)	26 (26.3)	

Data are presented as ‘count (%)’ of patients unless otherwise indicated.

a Because <2% of the sample fell into a racial category other than non-Hispanic White or non-Hispanic Black, we grouped non-White patients into a single group composed primarily (but not exclusively) of Black patients. SD: standard deviation, HNC: head and neck cancer, HPV: human papillomavirus.

**Table 2•:** Survival status by quintile.

	Median survival (IQR) months	End of study survival status (count, %)	
		Living	Deceased/lost to follow-up
Q1	83.34 (33.75, 106.68)	4 (57.1)	3 (42.9)
Q2	64.11 (32.15, 89.56)	55 (75.3)	18 (24.7)
Q2	60.59 (30.61, 96.55)	79 (71.8)	31 (28.2)
Q4	53.33 (28.82, 83.61)	90 (59.6)	61 (40.4)
Q5	51.78 (21.63, 79.89)	49 (49.5)	50 (50.5)
*p*-value	0.156	0.002	

IQR: inter-quartile range.

**Table 3•:** Community-level lifestyle interventions for HNC survivors.

Domain	Intervention	Description/example
Nutrition and Food Access	Community gardens, mobile produce markets	Increase access to fresh produce in food deserts
	Culturally tailored nutrition education	Memorial Sloan Kettering’s Global Menus for cancer patients
	Food bank partnerships	Provide cancer-friendly meal kits
Physical Activity	Free/low-cost exercise programs	Community center-based fitness tailored for survivors
	Walking groups, neighborhood fitness challenges	Promote physical activity and social cohesion
Sleep and Stress	Mindfulness, yoga classes	Offered via libraries, churches, or community centers
	Behavioral health integration	Address insomnia, anxiety, depression
Social Support	Peer mentorship programs	Cleveland Clinics 4th Angel Mentoring Program
	Community-based survivorship clinics	Navigation, psychosocial support
Health Literacy	Cancer advocacy training	Train residents as health navigators
	Mobile survivorship units	Pop-up clinics for follow-up care
Risk Behavior Reduction	Tobacco cessation programs	Community-based cessation
	Alcohol use reduction campaigns	Tailored messaging for HNC populations
Access and Transportation	Non-emergency medical transportation	Support for follow-up visits and rehab
	Telehealth survivorship services	Overcome geographic and mobility barriers
Policy and Environment	Built environment improvements	Safe walking paths, lighting, green spaces
	Local policy advocacy	Zoning for healthy food retail and recreational areas

## Data Availability

The data supporting the findings of this publication can be made available upon request.
